# Analysis of high fat diet induced genes during mammary gland development: identifying role players in poor prognosis of breast cancer

**DOI:** 10.1186/1756-0500-7-543

**Published:** 2014-08-18

**Authors:** Raquel C Martinez-Chacin, Megan Keniry, Robert K Dearth

**Affiliations:** Department of Biology, University of Texas-Pan American, 1201 West University Drive, Edinburg, TX 78539 USA; Department of Biology, University of Texas-Pan American, 1201 West University Drive, Edinburg, Texas 78539-2999 USA

**Keywords:** Breast cancer, Basal-like breast cancer, High fat diet, Poor prognosis, Mammary gland development, Diet-induced genes

## Abstract

**Background:**

Epidemiological studies have shown that consumption of a high-fat diet (HFD) increases the risk of developing breast cancer (BC). Studies in rodents have shown HFD causes changes in the genetic programming of the maturing mammary gland (MG) increasing the susceptibility of developing the disease. Less is known about how HFD induced genes impact BC development. HFD exposure two weeks before conception to six weeks of age was previously shown to dramatically change MG gene expression in 10 week old mice. Therefore, we investigated these differentially expressed HFD-induced genes for their expression in BC using the NKI 295 breast tumor dataset.

**Results:**

To examine the potential role of HFD induced genes in BC, we first investigated whether these HFD-induced genes in mouse MGs were differentially expressed in different types of human BC. Of the 28 HFD induced genes that were differentially expressed between BC subtypes in the NKI set, 79% were significantly higher in basal-like BC. Next, we analyzed whether HFD induced genes were associated with BC prognosis utilizing gene expression and survival data for each HFD induced gene from the NKI data and constructed Kaplan Meier survival plots. Significantly, 93% of the prognosis associated genes (13/14) were associated with poor prognosis (*P* = 0.002). Kaplan Meier analysis with 249 non-basal-like BC found that all but one of the genes examined were still significantly associated with poor prognosis. Furthermore, gene set enrichment analysis (GSEA) with HFD microarray data revealed that invasive BC genes where enriched in HFD samples that also had lost expression of luminal genes.

**Conclusions:**

HFD exposed mouse MGs maintain differential expression of genes that are found highly expressed in basal-like breast cancer. These HFD-induced genes associate with poor survival in numerous BC subtypes, making them more likely to directly impact prognosis. Furthermore, HFD exposure leads to a loss in the expression of luminal genes and a gain in expression of mesenchymal and BC invasion genes in MGs. Collectively, our study suggests that HFD exposure during development induces genes associated with poor prognosis, thus identifying how HFD diet may regulate BC development.

**Electronic supplementary material:**

The online version of this article (doi:10.1186/1756-0500-7-543) contains supplementary material, which is available to authorized users.

## Background

Recent evidence suggests that dietary content is one causal lifestyle factor that may contribute to breast cancer (BC) development. Studies focusing on migrant populations have shown that women migrating from low (Asian, Latin American) to high BC incidence rate countries (United States and other Western countries) acquire a higher risk for BC similar to those in the new country [1–6]. These studies suggest that dietary changes might be a strong contributing factor to the increased incidence rates observed in these migrating populations. A study analyzing the effects of dietary patterns and the risk of BC in women of different ethnicities concluded that women consuming a Western diet had a higher risk of BC [7]. The Western diet consists of foods primarily high in fat. A higher intake of dietary fat has been shown to increase BC risk in adults [8, 9].

A high-fat diet (HFD) may also determine the type of BC that will develop translating to a more precise treatment strategy increasing patient survival. Breast cancer tumors have been previously classified into subtypes based on their unique molecular/genetic expression profiles that correlate with phenotypic characteristics and clinical outcome [10–13]. The expression of estrogen receptor (ER) is a distinguishing marker between these tumors subtypes in combination with other molecular cues. Luminal subtype A (luminal A) and luminal subtype B (luminal B) are tumors that have high expression levels of ER (ER+) and are associated with a favorable prognosis. *ERBB2* is overexpressed in HER2 positive breast cancers whereas basal epithelial-like (basal-like) tumors are ER negative (ER-). HER2 and basal-like tumors are associated with a poor prognosis [10, 11, 13, 14]. Strikingly, the five year survival rate for patients with luminal A is 90% compared to as low as a 30% five year survival rate for patients with HER2 and basal-like BC tumors [14–16]. This difference is due, in part, to the difficulty in predicting a clinical course, tumor stage at diagnosis and availability of targeted therapy [14, 17]. Obesity significantly increases the risk of developing basal-like BC in premenopausal women, predicting a poor outcome in these individuals [18]. Commensurately, obese individuals are much less likely to develop luminal BC. Therefore, identifying the impact a HFD has on the type of BC a women develops may be critical in identifying the disease early to help predict a clinical course of action that would increase that patient’s survival.

The impact that HFD has on developing BC in humans has yet to be fully elucidated, in part, due to the limited number of human studies (and ability) to adequately assess the effect diet has on genetic regulation of the developing mammary gland (MG). Studies using rodent models have provided the most compelling evidence linking developmental exposure to a HFD to breast carcinogenesis [19–21]. These studies culminate to identify several theories to how consumption of a HFD during pre and/or postnatal development may cause BC including genetic reprogramming of the MG [22–25]. However, it is unclear as to how HFD might contribute to the development of specific types of BC correlating to the severity of the disease and patient survival. Thus, identifying MG HFD-induced transcriptional programs that are pertinent to specific types of human BC could have a tremendous impact on understanding the etiology of diet induced BC.

The purpose of this study was to examine genes induced by exposure to a HFD during MG development in the mouse for the role they may play in human BC. Luitjen *et. al.* [25] previously identified genes that were differentially expressed in 10 week old MGs after developmental HFD exposure. Strikingly, these changes were sustained even after HFD exposure, suggesting a long-term effect. To investigate how HFD may influence the development of BC, we examined the experimentally identified HFD-induced genes from the study by Luitjen *et al.* [25] in human breast tumors. The expression of HFD- induced genes was examined in BC by utilizing the widely studied NKI BC dataset from the Netherlands Cancer Institute, which contains gene expression and patient follow up/survival data from 295 women between the ages of 26 and 53 [10]. The NKI BC data set had been previously classified into five breast cancer subtypes [10]. Using Kaplan Meier analysis and gene set enrichment analysis (GSEA) we found that HFD induced genes were associated with poor prognosis and invasive BC. We also found that HFD leads to a loss in luminal gene expression and a gain in basal-like gene expression. Our data shows for the first time that HFD-induced genes are highly expressed in BCs that are of the basal-like subtype and are associated with poor prognosis.

## Methods

### Collection of HFD-induced genes and gene expression analysis in BC subtypes

Microarray data of differentially expressed genes was obtained from a study analyzing the effects of early diet on the genetic programming of the MG in wild-type mice [25]. As described previously [25], FVB wild-type mice were fed a HFD diet consisting of either a 24% high fat content of either n-6 (corn oils) or n-3 (flaxseed oil) PUFAs (polyunsaturated fatty acids) from two weeks prior conception to 6 weeks of age. Control animals were fed standard rodent chow (5% fat) during the same period. Gene expression profiling of the MG was performed at 10 weeks of age. Luitjen *et al.* [25] utilized the data from both types of fat (n-3 and n-6) in ANOVA analysis to identify differentially expressed genes. We examined the top one hundred differentially expressed genes that were induced by HFD with a *p*-value of 0.0001 or less based on the Luitjen ANOVA analysis for representation in the NKI dataset. The NKI dataset contains gene expression and survival data for 295 human breast tumors; these samples have also been previously classified based on breast cancer subtypes (basal-like = 46 tumors; HER2 positive = 49 tumors; luminal A = 88 tumors; luminal B = 81 tumors; normal-like = 31 tumors) [10, 11]. Our analysis focused on the HFD-induced genes found in the NKI data set (41 total). The average expression of each HFD-induced gene was determined for each BC sub-type. In order to determine whether HFD genes were differentially expressed between breast cancer subtypes, we performed the two tiered, type two T-test to determine whether each gene was expressed signficantly different between the basal-like subtype and the other sub-types of BC (HER2, luminal A, luminal B and normal-like). The T-test gave the same *p*-value as one way ANOVA, however a T-test anaylsis was used given we were only examining a single factor between two groups at a time. The false discovery rate (FDR) was calculated for differentially expressed genes to correct for multiple hypothesis testing. Changes in gene expression between subtypes that had an FDR of 0.01 or less were considered to be signifcant. It is improtant to note that the data sets utilized in this article were from experiemnts previously approved by their respective institutial ethics commitees and follow their national legeslation [10, 25].

### Survival analysis

A Kaplan Meier survival analysis for each HFD induced gene was performed using the NKI dataset and MedCalc software (Mariakerke, Belgium) for each gene to be analyzed. The association with prognosis of human breast cancer for each gene was obtained from this analysis: poor prognosis; good prognosis; no association. 295 breast tumors were divided into two equal-sized groups based on the expression of each gene; group one represented low expression of the gene and group two represented high expression of the gene. All availabe time points were utilized. P-values of less than 0.05 were considered significant associations. Based on the Chang *et al.* [11] classification of the NKI database, we utilized 249 breast cancer samples for the non-basal like breast tumor analysis.

### Additional statistical analysis of poor prognosis genes

HFD-induced genes that were associated with poor prognosis were analyzed as a group for overall differential expression between BC subtypes using NKI data. We performed ONE-WAY ANOVA with a Dunnett’s post test to determine the significance of mean gene expression between BC subtypes using the basal-like subtype as the reference group. Probability values of less than 0.05 were considered significant. The PC IBM INSTAT and PRISM software were used for the statistical analyses and graphs, respectively (GraphPad, San Diego, CA, USA).

### Gene set enrichment analysis

Gene set enrichment analysis (GSEA) was performed using the Broad Institute platform [26, 27]. Samples were analyzed with weighted, Signal2Noise default settings. Microarray gene expression data from Luitjen *et al*. was grouped based on diet (control low fat diet or HFD samples) [25]. Both types of HFD (n-3 and n-6 as indicated) were independently studied by GSEA with similar results. (Additional file 1: Table S1) contains the list of 361 breast cancer gene sets from the Molecular Signatures database, Broad Institute, that were utilized for analysis. The luminal gene sets utilized in GSEA are in (Additional file 2: Table S2).

## Results

### HFD during MG development induces basal-like BC genes

Murine MGs exposed to HFD during development (two weeks before conception to 6 weeks post birth) had dramatic changes in gene expression at ten weeks of age [25]. In order to investigate how HFD may influence BC development, we examined genes from the Luitjen *et al.* study that were induced by HFD with a *p*-value of 0.0001 or less for expression in BC subtypes by utilizing data for these genes in the NKI BC dataset. Gene expression signatures classify breast cancer into 5 basic subtypes: basal-like, HER2, luminal A, luminal B and normal-like. The basal-like subtype is associated with the worst prognosis whereas the luminal A is associated with the best prognosis [12, 13]. The average expression of each HFD induced gene was determined for each BC subtype (Table 1). Changes with an FDR of 0.01 or less were considered significantly different. Twenty-eight out of the 41 HFD induced genes showed statistically different levels of gene expression between the luminal A (associated with good prognosis) and basal-like (associated with poor prognosis) subtypes of BC; 79% of these genes were significantly higher in basal-like BC compared to luminal A BC. Therefore, HFD exposed glands preferentially maintain induced expression of genes that are found highly expressed in basal-like BC.

**Table 1:** **Expression of HFD-induced genes in breast cancer subtypes and association with breast cancer prognosis**

Gene ^a^	Basal-like ^b^	HER2 ^c^	Luminal A ^d^	Luminal B ^e^	Normal-like ^f^	Survival association ^g^
*MMP12*	0.17	-0.13*	-0.24*	-0.19*	-0.33*	Poor prognosis
*GPNMB*	-0.01	0.00	-0.18*	-0.06	-0.11	Poor prognosis
*CTSL*	0.07	0.04	-0.16*	-0.03*	-0.18*	Poor prognosis
*ITGAX*	0.00	0.07*	0.04	0.05	0.05	-
*LILRB4*	0.10	0.02	-0.15*	-0.04*	-0.22*	Poor prognosis
*TM7SF1*	-0.18	0.05*	0.05*	0.01*	-0.03*	-
*DNMT3A*	0.09	0.03	-0.08*	0.00*	-0.10*	Poor prognosis
*TYROBP*	0.06	0.01	-0.05*	-0.01	-0.13*	-
*DIO1*	-0.21	-0.18	-0.04*	-0.01*	-0.07*	-
*SLC11A1*	0.14	0.01*	-0.10*	-0.03*	-0.15*	Poor prognosis
*GALNS*	0.05	0.03*	0.01	0.03	0.01	-
*NCF2*	0.11	0.01*	-0.15*	-0.04*	-0.16*	Poor prognosis
*PTPNS1*	0.23	0.00*	-0.15*	-0.08*	-0.14*	Poor prognosis
*LGALS3*	-0.07	0.04*	-0.03	-0.01*	0.04*	-
*MTHFS*	-0.02	0.00	0.02	0.05*	-0.07	-
*IL1RN*	0.00	-0.17*	-0.13*	-0.11	-0.05	-
*CTSS*	0.12	-0.04*	-0.17*	-0.06*	-0.20*	-
*WBSCR5*	0.03	0.01	0.00	0.00	-0.06*	-
*PRKCD*	-0.05	0.02*	0.03*	0.04*	-0.02	-
*CAPG*	0.09	-0.03*	-0.06*	0.01*	-0.07*	-
*CD68*	0.06	0.09	-0.02*	0.02	-0.01*	-
*ITGB2*	0.11	0.04	-0.09*	-0.03*	-0.11*	Poor prognosis
*CSTB*	0.18	0.02*	-0.04*	-0.03*	-0.07*	Poor prognosis
*ATF3*	0.01	-0.13*	-0.12*	-0.09*	0.01	-
*CSF2RA*	0.01	-0.01	-0.02	-0.01	-0.11*	-
*WFS1*	-0.20	0.00*	0.03*	0.06*	-0.02*	-
*PPGB1*	0.00	0.07*	-0.05*	0.02	-0.07*	Poor prognosis
*ADAM8*	0.12	0.02*	-0.09*	-0.06*	-0.25*	Poor prognosis
*CCRL2*	0.06	0.07	-0.01	0.02	-0.06*	-
*VTN*	0.00	0.00	0.01	0.01	0.06	-
*RGS1*	0.04	0.01	-0.03*	-0.04*	-0.13*	-
*FCGR3*	0.09	0.06	-0.03*	0.03*	-0.08*	Poor prognosis
*EMR1*	0.06	-0.03	0.01	0.00*	0.03	-
*ARPC2*	0.06	0.03	-0.06*	-0.03*	-0.03*	-
*ESD*	-0.04	-0.04	0.02*	-0.01	0.08*	Good prognosis
*VAMP4*	0.06	0.05	0.05	0.05	-0.03	-
*CDKN1C*	-0.05	-0.05	-0.05	-0.12	0.13*	-
*HEXB*	-0.04	0.00	-0.02	-0.02	-0.03	-
*NAGLU*	-0.01	0.05*	0.02	0.04*	0.12*	-
*KCNH2*	0.00	-0.29*	-0.21*	-0.15*	-0.32*	-
*MSTF1*	0.02	0.03	0.04*	0.04*	0.04	-

^a^HFD induced genes (Luitjen *et al*.) that were present in the NKI human breast cancer data set as described in the *Methods*. ^b-f^Average gene expression among 5 different human breast cancer subtypes for these genes (Tumors: basal-like n = 46; HER2 n = 49; luminal A n = 88; luminal B n = 81; normal-like n = 31). ^g^Total of 13 genes associated with poor prognosis and 1 gene associated with good prognosis based on Kaplan Meier survival analysis for the indicated gene with 295 BC samples from the NKI dataset as described in the *Materials and Methods*. The plots for the genes that were significantly associated with prognosis are depicted in Figure 1 of this manuscript. *Gene expression statistically different with respect to basal-like subtype (False discovery Rate [FDR] < 0.01).

### HFD induced genes are strongly associated with poor prognosis in BC

**Figure 1:**
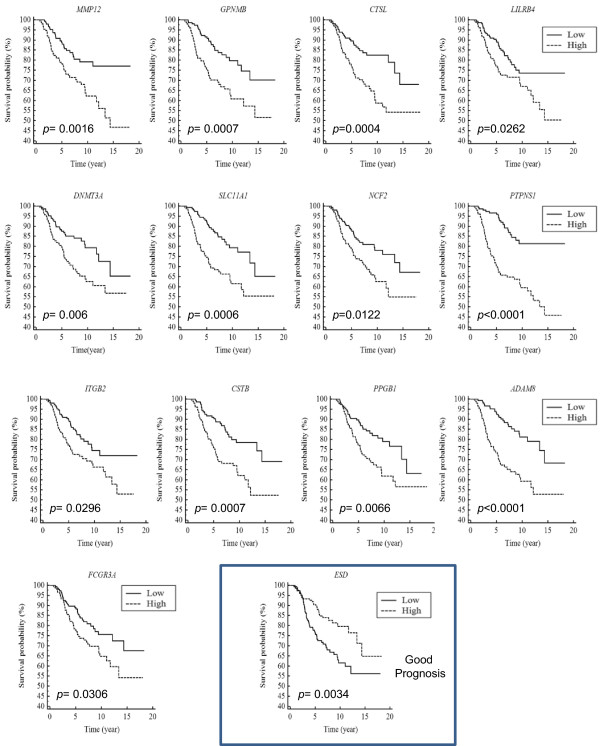
**HFD-induced genes are strongly associated with poor BC prognosis.** HFD induced genes from mouse MGs were examined for an association with human BC prognosis using Kaplan Meier survival analysis. Gene expression and survival data was obtained from the NKI (Netherlands Cancer Institute) dataset for each HFD induced gene. 295 breast tumors were divided into two equal-sized groups based on the expression of the indicated gene; one group represented low expression of the gene and the other group represented high expression of the gene. Depicted are 14 out of the 41 analyzed HFD induced genes; only these 14 genes showed significant association with breast cancer prognosis. 13 out of 14 HFD induced genes were significantly associated with a poor prognosis.

In order to examine whether HFD induced genes were associated with BC prognosis, we performed Kaplan Meier survival analysis using HFD induced genes from 10-week old mammary fat pads and the NKI dataset, as described in the Methods. A total of fourteen genes out of forty-one were associated with prognosis in BC (*p* < 0.05). Only one HFD-induced gene was associated with good prognosis; the high expression of *Esterase D* (ESD)*,* showed a higher survival probability when compared to the low gene expression (Figure 1, Table 1). Significantly, 93% of the prognosis associated genes (13 out of 14 HFD-induced genes) were associated with poor prognosis; the high expression of these genes showed a lower survival probability when compared to their low expression (Figure 1, Table 1) (*P* = 0.002). The AmiGO database [28] was utilized to identify gene ontologies that were associated with the HFD-induced poor prognosis genes. We found that the poor prognosis genes were associated with cell adhesion, proteolysis, immunological response, ion transport and DNA methylation (Table 2). Altogether, HFD-induced genes during mammary development are highly associated with poor survival in BC.

**Table 2:** **Cellular processes of genes associated with poor prognosis**

Gene	Description	GO reference	Gene ontology
*Mmp12*	Matrix metallopeptidase 12	GO:0006508	Proteolysis
*Gpnmb*	Glycoprotein (transmembrane)	GO: 0007155	Cell adhesion
*Ctsl*	Cathepsin L	GO:0006508	Proteolysis
*Lilrb4*	Leukocyte immunoglobulin-like receptor, subfamily B, member 4	GO: 0045671	Negative regulation of osteoclast differentiation
*Dnmt3a*	DNA (cytosine-5-)-methyltransferase 3 alpha	GO:0006306	DNA methylation
*Slc11a1*	Solute carrier family 11 (proton-coupled divalent metal ion transporter), member 1	GO:0070839	Divalent metal ion export
*Ncf2*	Neutrophil cytosolic factor 2	GO:0016175	Superoxide-generating NADPH oxidase activity
*Ptpns1*	Signal-regulatory protein alpha	GO: 0007155	Cell adhesion
*Itgb2*	Integrin, beta 2 (complement component 3 receptor 3 and 4 subunit)	GO:0007155	Cell adhesion
*Cstb*	Cystatin B (stefin B)	GO:0010466	Negative regulation of peptidase activity
*Ppgb1*	Cathepsin A	GO: 0006508	Proteolysis
*Adam8*	ADAM metallopeptidase domain 8	GO: 0006508	Proteolysis
*Fcgr3*	Fc fragment of IgG, low affinity IIIa, receptor (CD16a)	GO:0001788	Antibody-dependent cellular cytotoxicity

Gene ontologies from the AMIGO database were obtained for HFD induced genes that were found to be associated with poor prognosis.

**Figure 2:**
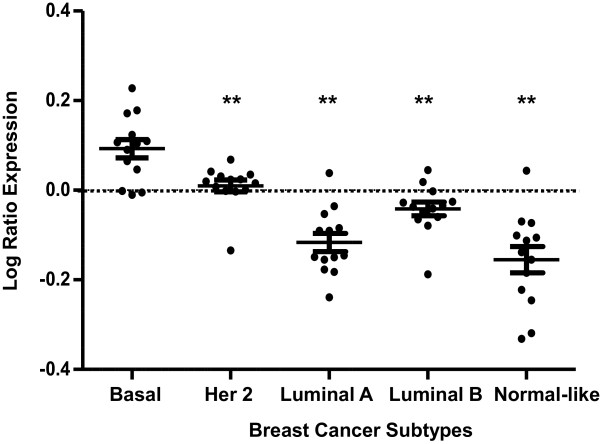
**Poor prognosis HFD genes are highly expressed in the basal-like subtype of BC.** We calculated the mean (±SEM) expression of each poor prognosis associated gene (from Figure 1) for each BC subtype using NKI gene expression data. Poor prognosis genes were significantly more highly expressed in the basal-like subtype compared to the other subtypes (Her2, luminal A, luminal B, and normal-like subtypes) based on ANOVA with Dunnett’s post-test using the basal-like subtype as the reference group. **p < 0.01.

The strong association between HFD-induced genes and poor prognosis in human breast cancer prompted us to further investigate this phenomena. We first examined the expression distribution of these genes in the 295 breast cancer tumors by plotting the expression of individual genes for each breast tumor sample, (Additional file 3: Figure S1) and (Additional file 4: Figure S2). One can see that there is a high expression of each poor prognosis associated gene in basal-like breast cancer samples whereas there is lower expression in luminal A breast cancers. Each of the poor prognosis genes is significantly less well expressed in luminal BC compared to basal-like BC, Table 1. Genes associated with poor prognosis were analyzed as a group for differential expression in human breast cancer subtypes. The average expression of these genes showed significant statistical difference in HER2, luminal A, luminal B, and normal-like subtypes with respect to the basal-like subtype (ANOVA p < 0.01, Figure 2). Of note, the differences in expression were more striking between basal-like BC samples and luminal A, luminal B and normal-like tumors, whereas a less dramatic, but still significant difference was found between basal-like tumors and HER2 positive tumors. This result is consistent with the fact that HER2 positive cancer are associated with poor prognosis as well [12, 14].

Although it is clear that HFD-induced genes are more strongly expressed in basal-like breast cancer, we were interested to examine whether these genes were associated with prognosis in other subtypes of breast cancer. To investigate this, we performed Kaplan Meier analysis with 249 non-basal-like NKI samples and found that all but 1 (92%) of the genes examined (Matrix metalloproteinase 12, *MMP12*) were still significantly associated with poor prognosis (Table 3). Therefore, HFD-induced genes associate with poor survival in a broad range of human breast cancer subtypes, making them likely to directly impact prognosis and have an influence on survival in many types of breast cancer.

**Table 3:** **Genes associated with poor prognosis in non-basal-like breast cancer**

Gene	Association	***P***-value
*GPNMB*	Poor prognosis	*P* = 0.0011
*CTSL*	Poor prognosis	*P* = 0.0022
*LILRB4*	Poor prognosis	*P* = 0.0274
*DNMT3A*	Poor prognosis	*P* = 0.0246
*SLC11A1*	Poor prognosis	*P* = 0.0461
*NCF2*	Poor prognosis	*P* = 0.0011
*PTPNS1*	Poor prognosis	*P* = 0.0008
*ITGB2*	Poor prognosis	*P* = 0.0013*
*CSTB*	Poor prognosis	*P* = 0.0332
*PPGB*	Poor prognosis	*P* = 0.0009
*ADAM8*	Poor prognosis	*P* = 0.0007
*FCGR3*	Poor prognosis	*P* = 0.0053
MMP12	-	*P* = 0.1092

Kaplan Meier analysis was performed with all non-basal breast cancer samples (249) from the NKI dataset and the HFD genes that were found to be associated with prognosis (from Figure 1 and Additional file 1: Table S1). *5 year Kaplan Meier curve was used instead of all time points for *ITGB2*. Strikingly, 12 out of 13 genes were still strongly associated with poor prognosis in non-basal breast cancer samples.

### HFD exposure induces BC mesenchymal and invasion genes

**Figure 3:**
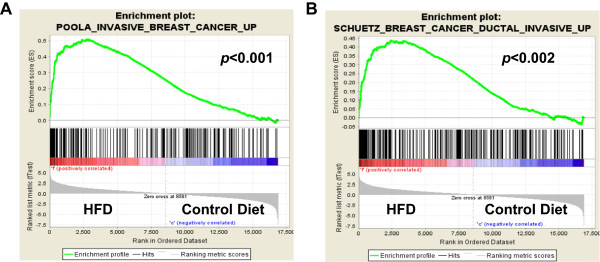
**BC invasion genes are enriched in HFD microarray samples.** Gene set enrichment analysis (GSEA) was performed with microarray data from 10 week old mice treated with HFD (labeled F) or control diet (labeled C) and 361 curated genes sets from the Molecular Signatures Database, Broad Institute; enrichment plots for two of the analyzed gene sets are shown. In these analyses, genes are ranked based on their association with the HFD phenotype or the control phenotype. Genes most strongly associated with HFD would be on the far right. Enrichment scores (ES) are calculated using a weighted Kolmogorov-Smirnov-style statistic. Lines underneath the graph depict the genes that are contained in the investigated gene set. **A-B**, genes associated with invasion in BC were highly associated with HFD samples. *P* values are indicated on plots.

HFD exposure during development in mice leads to dramatic changes in mammary fat pad gene expression. These changes strongly suggest that the breast tissue exposed to HFD are developmentally altered and may sustainably express programs that parallel those found in certain types of BC. To investigate this, we performed GSEA with 361 curated BC gene sets from the Broad Institute Molecular Signatures Database. Each of the 361 examined gene sets contained genes that were previously determined to be differentially expressed under defined condition in breast cancer. In GSEA, Kolmogorov-Smirnov-style statistics are employed to investigate whether gene sets are more highly expressed (enriched) between two conditions in microarray analyses. We compared microarray gene expression data from control low fat MGs to data from HFD MGs to determine whether any of the BC gene sets were significantly enriched in HFD samples. We found that genes associated with BC invasion were significantly enriched in HFD samples, Figure 3 and Table 4. Enrichment plots are depicted in Figure 3 in which genes are ranked based on their association with given phenotypes, in this case the phenotypes are HFD (on the right) or control diet (on the left). The lines underneath the enrichment plot depict genes that are contained on the gene list being investigated. One can see a striking enrichment of invasion genes in HFD treated samples, Figure 3. Therefore, HFD leads to an increase in the expression of genes involved in BC invasion.

**Table 4:** **Breast cancer gene sets that are enriched in HFD microarray samples**

GENE SET NAME	SIZE	ES	NES	NOM
				***p***-value
SMID_BREAST_CANCER_NORMAL_LIKE_UP	348	0.50	2.13	<0.001
POOLA_INVASIVE_BREAST_CANCER_UP	215	0.51	1.96	<0.001
BERTUCCI_MEDULLARY_VS_DUCTAL_BREAST_CANCER_UP	142	0.46	1.91	<0.001
FINAK_BREAST_CANCER_SDPP_SIGNATURE	21	0.65	1.87	<0.001
FINETTI_BREAST_CANCER_KINOME_GREEN	15	0.75	1.86	<0.001
SCHUETZ_BREAST_CANCER_DUCTAL_INVASIVE_UP	266	0.44	1.77	<0.001
SMID_BREAST_CANCER_LUMINAL_B_DN	412	0.36	1.69	<0.001
JOHNSTONE_PARVB_TARGETS_3_UP	306	0.34	1.64	<0.001

Gene set enrichment analysis was preformed with microarray data from high fat and low fat diet as described in the *Materials and Methods* and 361 curated breast cancer gene sets on the Molecular Signatures Database, Broad Institute. In GSEA enrichment scores (ES) are calculated for each gene set using a Kolmogorov-Smirnov-style statistic. The ES indicates how strongly associated a gene set is with a given phenotype or not. GSEA generates nominal *p*-values (NOM *p*-value) using on a phenotype based permutation comparing ES with a null distribution. Normalized enrichment scores (NES) are adjusted enrichment scores based on the number of genes in each examined set. Gene sets with a nominal P-value (NOM p-value) of 0.001 or less and a normalized enrichment score (NES) of 1.5 or greater are shown. These data suggest that common genes are expressed in mammary fat pads from mice treated with HFD and invasive breast cancer.

**Figure 4:**
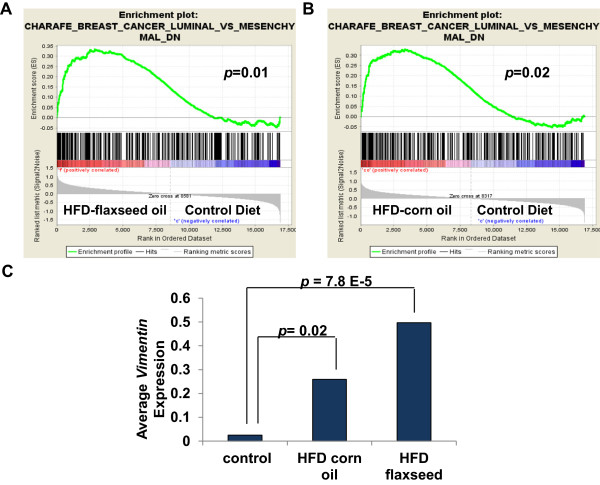
**BC Mesenchymal genes are enriched in HFD microarray samples.** GSEA was performed with microarray data from 10 week old mice that were fed HFD (labeled F for flaxseed or CO for corn oil) or normal diet (labeled C for control) and 361 curated gene sets from the Molecular Signatures Database, Broad Institute; enrichment plots are shown for the Charafe_Breast_Cancer_Luminal_VS_Mesenchymal_DN geneset. **A-B**, genes associated with mesenchymal characteristics in BC were significantly associated with the HFD samples. **C**, the average expression of the mesenchymal marker *Vimentin* was calculated for HFD (corn oil or flaxseed) and control samples using data from the Luitjen *et al.* microarray data set. *Vimentin* was strikingly expressed 10–20 fold higher in HFD treated MG compared to control MGs. *P* values are indicated on graphs.

To further examine HFD mediated changes in gene expression in the MG with regard to BC development, we performed GSEA with 53 luminal BC gene sets from the Molecular Signatures database, Broad Institute. We found that genes that are normally down-regulated in the luminal subtype of BC (and are highly expressed in mesenchymal BC) were enriched in HFD treated samples, suggesting that these mice may have altered breast development to a more basal phenotype (Figure 4 A-B, Table 5). Enrichment plots for analysis with gene sets that are down in luminal BC and high in mesenchymal BC (Charafe_luminal_versus_mesenchymal_DN) indicate a clear increase in mesenchymal BC genes upon HFD treatment, Figure 4. Furthermore, we found that stromal genes from poor prognosis BC (gene-set FINAK_BREAST_CANCER_SDPP_SIGNATURE, SDPP is an abbreviation for stroma-derived prognostic predictor of BC disease outcome), were strongly associated with HFD MGs with a Normalized Enrichment Score (NES) of 1.87, *p* = <0.001 (Table 4). One of the top enriched genes in our GSEA was the mesenchymal marker *Vimentin*. We assessed the average *Vimentin* expression in control MGs versus HFD fed MGs using the Luitjen *et al.* [25] microarray dataset and found that HFD exposed MGs expressed significantly higher levels of the basal-like BC marker *Vimentin.* The average expression of *Vimentin* was strikingly tenfold higher with n-3 HFD and 20 fold higher with n-6 HFD (Figure 4C). In sum, HFD exposure leads to a loss in the expression of luminal genes and a gain in expression of mesenchymal and BC invasion genes in MGs.

**Table 5:** **HFD leads to a loss in the expression of luminal genes**

GENE SET NAME	Size	ES	NES	NOM
				***p***-value
SMID_BREAST_CANCER_NORMAL_LIKE_UP	348	0.50	2.09	<0.001
BERTUCCI_MEDULLARY_VS_DUCTAL_BREAST_CANCER_UP	142	0.47	1.95	<0.001
FINETTI_BREAST_CANCER_KINOME_GREEN	15	0.75	1.84	0.01
SMID_BREAST_CANCER_LUMINAL_B_DN	412	0.37	1.70	<0.001
FARMER_BREAST_CANCER_CLUSTER_1	25	0.66	1.70	0.01
CHARAFE_BREAST_CANCER_LUMINAL_VS_MESENCHYMAL_DN	325	0.33	1.53	0.01
HOWLIN_PUBERTAL_MAMMARY_GLAND	60	0.40	1.50	0.03

Gene set enrichment analysis was performed with 53 gene sets containing luminal genes in breast cancer denoted (form the Molecular Signatures Database, Broad Institute). In GSEA enrichment scores (ES) are calculated for each gene set using a Kolmogorov-Smirnov-style statistic. The ES indicates how strongly associated a gene set is with a given phenotype or not. GSEA generates nominal *p*-values (NOM *p*-value) using on a phenotype based permutation comparing ES with a null distribution. Normalized enrichment scores (NES) are adjusted enrichment scores based on the number of genes in each examined set. We found that gene sets with down-regulation of luminal genes were enriched in the HFD samples.

## Discussion

BC is a heterogeneous disease. Identifying dietary factors that contribute to the development of different BC subtypes is important for the prevention and treatment of the disease. A HFD has been shown to increase BC risk in humans [8, 9] and several studies using rodent models have shown that exposure to a HFD *in utero* and/or during postnatal development significantly increase carcinogen stimulated MG tumorigenesis [19–21]. However, the developmental processes in the MG impacted by a HFD that mediate the heterogeneity of BC are unclear. Recently, work by Luitjen *et al.* [25] revealed that HFD exposure during MG development leads to sustained changes in gene expression. In the present study, we found that genes induced by a HFD in the mouse developing MG were strongly expressed in the human basal-like subtype of BC, suggesting that HFD exposed MGs may have abnormal basal-like characteristics. In line with this, we found increased expression of mesenchymal genes by GSEA including the well described marker *Vimentin* in HFD gland samples. Our analysis reveals that HFD exposure during development leads to an increase in basal-like characteristics in MGs along with a concomitant loss of luminal gene expression (Figures 3–4), which may promote an increase in basal-like BC development. This pattern of gene expression parallels the increase in basal-like BC/decrease in luminal BC that is found in obese women [18]. It remains to be determined as to which HFD exposed cells express the basal-like genes and whether the MG architecture is dramatically altered in a sustained manner in these models and in humans. Nonetheless, HFD profoundly affects MG gene expression in a sustained way, inducing genes that are expressed in basal-like BC.

Here we show that of the HFD-induced genes that were significantly associated with prognosis in BC, a striking 93% was significantly associated with poor prognosis. All but one these genes were still strongly associated with poor prognosis in the non-basal-like set of BCs (249 tumors examined). Therefore, the association between HFD-induced genes and prognosis in BC is highly compelling and spans many subtypes. While some skepticism could be drawn due to the relevance of cross-species analysis, a strong body of evidence has demonstrated the parallels between rodent and human MG structures, MG development and carcinogenesis [29–33]. Furthermore, mouse MG tumors have been shown to replicate the diversity of human BCs including tumor initiation, hormone dependency and histopathology across multiple MG endpoints [31–33]. Thus, our data suggests a model in which HFD impacts MG development to promote poor prognosis cancer.

Interestingly, we provide evidence that a HFD, regardless of content (n-3 or n-6 PUFAs), may preferentially promote basal-like BC. Epidemiological evidence supports the idea that a HFD increases BC risk. Whether this risk is a consequence of overall percentage of fat in an individual’s diet or type of PUFAs in the diet has long been debated. Numerous rodent studies have investigated the dietary impact of (n-3) and (n-6) PUFAs on BC risk. While rodent studies support the notion that diets high in (n-6) PUFAs increase breast carcinogenesis [34–37], diets primarily consisting of n-3 PUFAs have been suggested to protect against BC [38, 39]. However, Hilakivi-Clarke *et al.* [22], showed that prepubertal rats exposed to a high (n-3) PUFA diet resulted in key biological changes within the MG reflecting an increased susceptibility to BC. Furthermore, studies in adult rats have shown that exposure to a diet high in (n-3) PUFAs do not inhibit carcinogen induced mammary tumorigenesis, but may promote it [40, 41]. While the timing of exposure could play a factor (fetal, adolescence, adult), human studies have yet to provide conclusive evidence that diets consisting of primarily (n-3) or (n-6) PUFAs play a profound role in increasing or decreasing BC risk. [42–44]. Thus, the influence of a HFD on BC development maybe more a consequence of the amount fat consumed regardless of the source and our data supports this hypothesis.

## Conclusions

Given the striking association between HFD-induced genes during development and prognosis in BC, it is imperative to examine these phenomena in humans. The greatest strides in decreasing cancer mortality have been made by prevention as with the link between smoking and lung cancer. It will be important to determine whether developmental HFD exposure as in this study and/or exposure in adults lead to the induction of poor prognosis genes. Also, it will be critical to determine whether these changes are permanent or require the presence of HFD or are maintained for only a certain period of time. It will also be crucial to determine how any of the maintained changes in gene expression are propagated. Based on the HFD mediated induction of DNA (cytosine-5-)-methyltransferase 3 alpha (*DNMT3A*), perhaps an epigenetic change is responsible for these sustained shifts in gene expression. *DNMT3A* functions in *de novo* methylation, is important in development and altered expression levels have been found in several different types of human cancers [45]. If the link between poor prognosis in breast cancer and developmental HFD exposure bare out in humans, efforts can be made to prevent such exposures. In addition, if this link holds true in human breast cancer, it may be possible to identify women who are more susceptible to poor prognosis cancers based on the expression of these newly identified HFD-induced basal-like characteristics such as the mesenchymal marker *Vimentin*. Importantly, vimentin has been shown to play a significant role in the epithelial-mesenchymal transition (EMT) process in BC [46, 47]. EMT is a cellular reprograming process that changes the shape of epithelial cells to exhibit a more motile mesenchymal phenotype (reviewed in [48]). *In vitro Vimentin* over expression in human BC cells has been shown to contribute to this process, thus increasing BC cell motility and invasive properties [47]. In human breast cancer samples, vimentin expression is found in high-grade ductal carcinomas with low ER expression levels [49]. Thus, identifying early changes in vimentin expression in breast epithelium due to HFD exposure might be used to predict clinical outcomes translating to better preventive treatment strategies.

In sum, we show that HFD induces the expression of genes that are associated with poor prognosis in BC. HFD exposed MGs showed high expression of the mesenchymal marker *Vimentin* as well as a loss in luminal markers. We propose that developmental HFD exposure leads to sustained changes in gene expression that promote the development of poor prognosis cancers including basal-like BC.

## Availability of supporting data

The data supporting the results of the article is included within the article and in supplementary materials.

## Electronic supplementary material

Additional file 1: **361 breast cancer gene sets that were utilized in GSEA.** This excel file details the 361 breast cancer gene sets from the Molecular Signatures Database that were utilized for gene set enrichment analysis with the HFD microarray data. NAME denotes the gene set name, original size denotes the number of genes in the curated gene set, after restricting to data set denotes the number of genes that were found on both the gene list and the HFD microarray data set, and status denotes whether the gene list was included in the analysis or not. A small number of gene lists were not included in the analysis, if there were not enough genes. (PDF 279 KB)

Additional file 2: **53 luminal breast cancer gene sets that were utilized in GSEA.** This excel file details the 53 breast cancer gene sets from the Molecular Signatures Database that were utilized for gene set enrichment analysis with the HFD microarray data. NAME denotes the set name, original size denotes the number of genes in the curated gene set, after restricting to data set denotes the number of genes that were found on both the gene list and the HFD microarray data set, and status denotes whether the gene list was included in the analysis or not. A small number of gene lists were not included in the analysis, if there were not enough genes. (PDF 184 KB)

Additional file 3: **Poor prognosis associated HFD-induced genes MMP12, GPNMB, CTSL and LILRB4 are highly expressed in basal-like BC.** Depicted are histograms with log ration expression values for the indicated gene for each tumor (from the 295 NKI breast tumor dataset). Tumors are grouped together based on subtype. These subtypes are indicated at the top of the figure: Basal-like tumors are 1–46, HER2 are 47–95, Luminal A are 96–183, Luminal B are 184–264 and Normal-like tumors are 265–295. (PDF 326 KB)

Additional file 4: **Poor prognosis associated HFD-induced genes DNMT3A, SLC11A1, NCF2 and PTPNS1 are highly expressed in basal-like BC.** Depicted are histograms with log ration expression values for the indicated gene for each tumor (from the 295 NKI breast tumor dataset). Tumors are grouped together based on subtype. These subtypes are indicated at the top of the figure: Basal-like tumors are 1–46, HER2 are 47–95, Luminal A are 96–183, Luminal B are 184–264 and Normal-like tumors are 265–295. (PDF 335 KB)

## Abbreviations

BC: Breast cancer
HFD: High fat diet
ER: Estrogen receptor
ERBB2/HER2: Epidermal growth factor receptor 2
MG: Mammary gland
GSEA: Gene set enrichment analysis
PUFAs: Polyunsaturated fatty acids
FDR: False discovery rate
ESD: Esterase D
MMPR: Matrix metalloproteinase 12
DNMT3A: DNA (cytosine-5-)-methyltransferase 3 alpha
EMT: Epithelial-mesenchymal transition
NCI: Netherlands cancer institute
NOM: Normal p-values
ES: Enrichment scores
NES: Normalized enrichment scores.
