# Supplementary material for: Analysis of high fat diet induced genes during mammary gland development: identifying role players in poor prognosis of breast cancer
Source: BMC Res Notes. 2014 Aug 18;7:543. doi: 10.1186/1756-0500-7-543 (PMC4153917; doi:10.1186/1756-0500-7-543)
Supplement: Supplementary file 1 — Additional file 1: 361 breast cancer gene sets that were utilized in GSEA. This excel file details the 361 breast cancer gene sets from the Molecular Signatures Database that were utilized for gene set enrichment analysis with the HFD microarray data. NAME denotes the gene set name, original size denotes the number of genes in the curated gene set, after restricting to data set denotes the number of genes that were found on both the gene list and the HFD microarray data set, and status denotes whether the gene list was included in the analysis or not. A small number of gene lists were not included in the analysis, if there were not enough genes. (PDF 279 KB) [file 13104_2014_3097_MOESM1_ESM.pdf]

Additional File 1. 361 Breast Cancer Gene Sets that were Utilized in GSEA

NAME	ORIGINAL SIZE	AFTER RESTRICTING TO DATASET	STATUS
AIGNER_ZEB1_TARGETS	35	28	
AIYAR_COBRA1_TARGETS_DN	29	22	
AIYAR_COBRA1_TARGETS_UP	39	31	
BAE_BRCA1_TARGETS_DN	32	29	
BAE_BRCA1_TARGETS_UP	75	64	
BCAT_BILD_ET_AL_DN	46	33	
BCAT_BILD_ET_AL_UP	49	32	
BECKER_TAMOXIFEN_RESISTANCE_DN	52	39	
BECKER_TAMOXIFEN_RESISTANCE_UP	50	44	
BENPORATH_CYCLING_GENES	648	457	
BENPORATH_EED_TARGETS	1062	712	
BENPORATH_ES_1	379	281	
BENPORATH_ES_2	40	25	
BENPORATH_ES_CORE_NINE	9	8	
BENPORATH_ES_CORE_NINE_CORRELATED	100	84	
BENPORATH_ES_WITH_H3K27ME3	1118	776	
BENPORATH_MYC_MAX_TARGETS	775	599	
BENPORATH_MYC_TARGETS_WITH_EBOX	230	197	
BENPORATH_NANOG_TARGETS	988	694	
BENPORATH_NOS_TARGETS	179	132	
BENPORATH_OCT4_TARGETS	290	214	
BENPORATH_PRC2_TARGETS	652	449	
BENPORATH_PROLIFERATION	147	118	
BENPORATH_SOX2_TARGETS	734	522	
BENPORATH_SUZ12_TARGETS	1038	698	
BERTUCCI_MEDULLARY_VS_DUCTAL_BREAST_CANCER_DN	169	119	
BERTUCCI_MEDULLARY_VS_DUCTAL_BREAST_CANCER_UP	206	142	
BHAT_ESR1_TARGETS_NOT_VIA_AKT1_DN	88	69	
BHAT_ESR1_TARGETS_NOT_VIA_AKT1_UP	211	147	
BHAT_ESR1_TARGETS_VIA_AKT1_DN	82	61	
BHAT_ESR1_TARGETS_VIA_AKT1_UP	281	187	
BHATI_G2M_ARREST_BY_2METHOXYESTRADIOL_DN	127	88	
BHATI_G2M_ARREST_BY_2METHOXYESTRADIOL_UP	125	86	
BIOCARTA_ATRBRCA_PATHWAY	21	18	
BIOCARTA_HER2_PATHWAY	22	20	
BIOCARTA_STATHMIN_PATHWAY	19	16	
CAFFAREL_RESPONSE_TO_THC_24HR_3_DN	13	10	
CAFFAREL_RESPONSE_TO_THC_24HR_3_UP	8	6	
CAFFAREL_RESPONSE_TO_THC_24HR_5_DN	59	45	
CAFFAREL_RESPONSE_TO_THC_24HR_5_UP	34	26	
CAFFAREL_RESPONSE_TO_THC_8HR_3_DN	10	7	
CAFFAREL_RESPONSE_TO_THC_8HR_3_UP	5		Rejected!
CAFFAREL_RESPONSE_TO_THC_8HR_5_DN	11	8	
CAFFAREL_RESPONSE_TO_THC_8HR_5_UP	16	10	
CAFFAREL_RESPONSE_TO_THC_DN	31	23	
CAFFAREL_RESPONSE_TO_THC_UP	33	25	
CHARAFE_BREAST_CANCER_BASAL_VS_MESENCHYMAL_DN	50	35	
CHARAFE_BREAST_CANCER_BASAL_VS_MESENCHYMAL_UP	121	76	
CHARAFE_BREAST_CANCER_LUMINAL_VS_BASAL_DN	455	337	
CHARAFE_BREAST_CANCER_LUMINAL_VS_BASAL_UP	380	230	
CHARAFE_BREAST_CANCER_LUMINAL_VS_MESENCHYMAL_DN	460	325	
CHARAFE_BREAST_CANCER_LUMINAL_VS_MESENCHYMAL_UP	450	293	
CHEN_HOXA5_TARGETS_6HR_DN	6		Rejected!
CHEN_HOXA5_TARGETS_6HR_UP	10	8	
CHEN_HOXA5_TARGETS_9HR_DN	41	31	
CHEN_HOXA5_TARGETS_9HR_UP	223	147	
CHENG_IMPRINTED_BY_ESTRADIOL	110	71	
CHIN_BREAST_CANCER_COPY_NUMBER_DN	16	11	
CHIN_BREAST_CANCER_COPY_NUMBER_UP	27	23	
CHUANG_OXIDATIVE_STRESS_RESPONSE_DN	11	10	
CHUANG_OXIDATIVE_STRESS_RESPONSE_UP	28	24	
CLIMENT_BREAST_CANCER_COPY_NUMBER_DN	8	6	
CLIMENT_BREAST_CANCER_COPY_NUMBER_UP	23	21	
CORDENONSI_YAP_CONSERVED_SIGNATURE	57	49	
CREIGHTON_AKT1_SIGNALING_VIA_MTOR_DN	23	18	
CREIGHTON_AKT1_SIGNALING_VIA_MTOR_UP	34	26	
CREIGHTON_ENDOCRINE_THERAPY_RESISTANCE_1	528	357	
CREIGHTON_ENDOCRINE_THERAPY_RESISTANCE_2	473	210	
CREIGHTON_ENDOCRINE_THERAPY_RESISTANCE_3	720	477	
CREIGHTON_ENDOCRINE_THERAPY_RESISTANCE_4	307	202	
CREIGHTON_ENDOCRINE_THERAPY_RESISTANCE_5	482	324	
DAIRKEE_CANCER_PRONE_RESPONSE_BPA	51	44	
DAIRKEE_CANCER_PRONE_RESPONSE_BPA_E2	118	97	
DAIRKEE_CANCER_PRONE_RESPONSE_E2	28	23	
DAIRKEE_TERT_TARGETS_DN	124	81	
DAIRKEE_TERT_TARGETS_UP	380	271	
DITTMER_PTHLH_TARGETS_DN	73	59	

DITTMER_PTHLH_TARGETS_UP	112	97
DOANE_BREAST_CANCER_CLASSES_DN	34	30
DOANE_BREAST_CANCER_CLASSES_UP	72	44
DOANE_BREAST_CANCER_ESR1_DN	48	41
DOANE_BREAST_CANCER_ESR1_UP	112	83
DOANE_RESPONSE_TO_ANDROGEN_DN	241	182
DOANE_RESPONSE_TO_ANDROGEN_UP	184	108
E2F3_UP.V1_DN	183	73
E2F3_UP.V1_UP	196	120
EINAV_INTERFERON_SIGNATURE_IN_CANCER	27	19
ENGELMANN_CANCER_PROGENITORS_DN	70	40
ENGELMANN_CANCER_PROGENITORS_UP	48	42
FARMER_BREAST_CANCER_APOCRINE_VS_BASAL	330	245
FARMER_BREAST_CANCER_APOCRINE_VS_LUMINAL	326	235
FARMER_BREAST_CANCER_BASAL_VS_LULMINAL	330	241
FARMER_BREAST_CANCER_CLUSTER_1	44	25
FARMER_BREAST_CANCER_CLUSTER_2	33	30
FARMER_BREAST_CANCER_CLUSTER_4	16	14
FARMER_BREAST_CANCER_CLUSTER_5	19	18
FARMER_BREAST_CANCER_CLUSTER_6	16	14
FARMER_BREAST_CANCER_CLUSTER_7	20	13
FARMER_BREAST_CANCER_CLUSTER_8	7	Rejected!
FINAK_BREAST_CANCER_SDPP_SIGNATURE	26	21
FINETTI_BREAST_CANCER_KINOME_GREEN	16	15
FINETTI_BREAST_CANCER_KINOME_RED	16	15
FINETTI_BREAST_CANCERS_KINOME_BLUE	21	17
FINETTI_BREAST_CANCERS_KINOME_GRAY	15	13
FOURNIER_ACINAR_DEVELOPMENT_EARLY_DN	6	Rejected!
FOURNIER_ACINAR_DEVELOPMENT_EARLY_UP	21	13
FOURNIER_ACINAR_DEVELOPMENT_LATE_2	277	216
FOURNIER_ACINAR_DEVELOPMENT_LATE_DN	21	19
FOURNIER_ACINAR_DEVELOPMENT_LATE_UP	11	9
FRASOR_RESPONSE_TO_ESTRADIOL_DN	82	65
FRASOR_RESPONSE_TO_ESTRADIOL_UP	37	32
FRASOR_RESPONSE_TO_SERM_OR_FULVESTRANT_DN	50	46
FRASOR_RESPONSE_TO_SERM_OR_FULVESTRANT_UP	24	14
FRASOR_TAMOXIFEN_RESPONSE_DN	11	8
FRASOR_TAMOXIFEN_RESPONSE_UP	51	38
FUJII_YBX1_TARGETS_DN	202	170
FUJII_YBX1_TARGETS_UP	43	31
GINESTIER_BREAST_CANCER_20Q13_AMPLIFICATION_DN	180	101
GINESTIER_BREAST_CANCER_20Q13_AMPLIFICATION_UP	119	79
GINESTIER_BREAST_CANCER_ZNF217_AMPLIFIED_DN	335	184
GINESTIER_BREAST_CANCER_ZNF217_AMPLIFIED_UP	78	51
GRAESSMANN_APOPTOSIS_BY_DOXORUBICIN_DN	1781	Rejected!
GRAESSMANN_APOPTOSIS_BY_DOXORUBICIN_UP	1142	861
GRAESSMANN_APOPTOSIS_BY_SERUM_DEPRIVATION_DN	234	182
GRAESSMANN_APOPTOSIS_BY_SERUM_DEPRIVATION_UP	552	422
GRAESSMANN_RESPONSE_TO_MC_AND_DOXORUBICIN_DN	770	558
GRAESSMANN_RESPONSE_TO_MC_AND_DOXORUBICIN_UP	612	470
GRAESSMANN_RESPONSE_TO_MC_AND_SERUM_DEPRIVATION_DN	84	66
GRAESSMANN_RESPONSE_TO_MC_AND_SERUM_DEPRIVATION_UP	211	167
GU_PDEF_TARGETS_DN	39	28
GU_PDEF_TARGETS_UP	71	64
GYORFFY_DOXORUBICIN_RESISTANCE	56	35
GYORFFY_MITOXANTRONE_RESISTANCE	57	36
HAN_SATB1_TARGETS_DN	442	319
HAN_SATB1_TARGETS_UP	395	274
HEDENFALK_BREAST_CANCER_BRACX_DN	20	15
HEDENFALK_BREAST_CANCER_BRACX_UP	20	14
HEDENFALK_BREAST_CANCER_BRCA1_VS_BRCA2	163	130
HEDENFALK_BREAST_CANCER_HEREDITARY_VS_SPORADIC	50	38
HERNANDEZ_ABERRANT_MITOSIS_BY_DOCETACEL_2NM_DN	25	21
HERNANDEZ_ABERRANT_MITOSIS_BY_DOCETACEL_2NM_UP	81	58
HERNANDEZ_ABERRANT_MITOSIS_BY_DOCETACEL_4NM_DN	7	7
HERNANDEZ_ABERRANT_MITOSIS_BY_DOCETACEL_4NM_UP	23	16
HERNANDEZ_MITOTIC_ARREST_BY_DOCETAXEL_1_DN	38	31
HERNANDEZ_MITOTIC_ARREST_BY_DOCETAXEL_1_UP	36	25
HERNANDEZ_MITOTIC_ARREST_BY_DOCETAXEL_2_DN	19	10
HERNANDEZ_MITOTIC_ARREST_BY_DOCETAXEL_2_UP	64	49
HESSON_TUMOR_SUPPRESSOR_CLUSTER_3P21_3	7	6
HOFFMAN_CLOCK_TARGETS_DN	10	8
HOFFMAN_CLOCK_TARGETS_UP	11	8
HONMA_DOCETAXEL_RESISTANCE	34	26
HONRADO_BREAST_CANCER_BRCA1_VS_BRCA2	18	16
HOOI_ST7_TARGETS_DN	123	67
HOOI_ST7_TARGETS_UP	94	56
HUANG_DASATINIB_RESISTANCE_DN	69	48
HUANG_DASATINIB_RESISTANCE_UP	81	63
HUPER_BREAST_BASAL_VS_LUMINAL_DN	59	43
HUPER_BREAST_BASAL_VS_LUMINAL_UP	54	38

IYENGAR_RESPONSE_TO_ADIPOCYTE_FACTORS	10	9
JAZAERI_BREAST_CANCER_BRCA1_VS_BRCA2_DN	43	34
JAZAERI_BREAST_CANCER_BRCA1_VS_BRCA2_UP	49	38
JOHNSTONE_PARVB_TARGETS_1_DN	63	42
JOHNSTONE_PARVB_TARGETS_1_UP	7	Rejected!
JOHNSTONE_PARVB_TARGETS_2_DN	336	228
JOHNSTONE_PARVB_TARGETS_2_UP	140	104
JOHNSTONE_PARVB_TARGETS_3_DN	918	602
JOHNSTONE_PARVB_TARGETS_3_UP	430	306
KEGG_HOMOLOGOUS_RECOMBINATION	28	23
KRAS.600.LUNG.BREAST_UP.V1_DN	289	186
KRAS.600.LUNG.BREAST_UP.V1_UP	288	183
KRAS.BREAST_UP.V1_DN	145	81
KRAS.BREAST_UP.V1_UP	146	92
KRAS.LUNG.BREAST_UP.V1_DN	145	92
KRAS.LUNG.BREAST_UP.V1_UP	145	95
LANDEMAINE_LUNG_METASTASIS	21	13
LANDIS_ERBB2_BREAST_PRENEOPLASTIC_DN	55	45
LANDIS_ERBB2_BREAST_PRENEOPLASTIC_UP	20	17
LANDIS_ERBB2_BREAST_TUMORS_324_DN	149	123
LANDIS_ERBB2_BREAST_TUMORS_324_UP	150	121
LANDIS_ERBB2_BREAST_TUMORS_65_DN	37	32
LANDIS_ERBB2_BREAST_TUMORS_65_UP	22	18
LE_SKI_TARGETS_DN	8	7
LE_SKI_TARGETS_UP	17	13
LIEN_BREAST_CARCINOMA_METAPLASTIC	35	32
LIEN_BREAST_CARCINOMA_METAPLASTIC_VS_DUCTAL_DN	114	65
LIEN_BREAST_CARCINOMA_METAPLASTIC_VS_DUCTAL_UP	83	63
LIU_BREAST_CANCER	30	19
LIU_COMMON_CANCER_GENES	79	43
LIU_LIVER_CANCER	38	26
LIU_NASOPHARYNGEAL_CARCINOMA	70	51
LIU_TOPBP1_TARGETS	16	9
MACLACHLAN_BRCA1_TARGETS_DN	16	14
MACLACHLAN_BRCA1_TARGETS_UP	21	17
MAEKAWA_ATF2_TARGETS	24	20
MARCHINI_TRABECTEDIN_RESISTANCE_DN	49	38
MARCHINI_TRABECTEDIN_RESISTANCE_UP	21	17
MASSARWEH_RESPONSE_TO_ESTRADIOL	61	44
MASSARWEH_TAMOXIFEN_RESISTANCE_DN	258	159
MASSARWEH_TAMOXIFEN_RESISTANCE_UP	578	375
MCBRYAN_PUBERTAL_BREAST_3_4WK_DN	39	30
MCBRYAN_PUBERTAL_BREAST_3_4WK_UP	214	172
MCBRYAN_PUBERTAL_BREAST_4_5WK_DN	196	158
MCBRYAN_PUBERTAL_BREAST_4_5WK_UP	271	228
MCBRYAN_PUBERTAL_BREAST_5_6WK_DN	137	98
MCBRYAN_PUBERTAL_BREAST_5_6WK_UP	116	99
MCBRYAN_PUBERTAL_BREAST_6_7WK_DN	79	68
MCBRYAN_PUBERTAL_BREAST_6_7WK_UP	197	151
MCBRYAN_PUBERTAL_TGFB1_TARGETS_DN	62	58
MCBRYAN_PUBERTAL_TGFB1_TARGETS_UP	169	151
MCBRYAN_TERMINAL_END_BUD_DN	7	Rejected!
MCBRYAN_TERMINAL_END_BUD_UP	12	10
MIKI_COEXPRESSED_WITH_CYP19A1	7	7
MISHRA_CARCINOMA_ASSOCIATED_FIBROBLAST_DN	24	19
MISHRA_CARCINOMA_ASSOCIATED_FIBROBLAST_UP	24	18
MORF_BRCA1	264	195
MYC_UP.V1_DN	182	78
MYC_UP.V1_UP	186	97
NADERI_BREAST_CANCER_PROGNOSIS_DN	18	17
NADERI_BREAST_CANCER_PROGNOSIS_UP	50	39
NAGASHIMA_EGF_SIGNALING_UP	58	49
NAGASHIMA_NRG1_SIGNALING_DN	58	36
NAGASHIMA_NRG1_SIGNALING_UP	176	149
NAM_FXYD5_TARGETS_DN	18	17
NIKOLSKY_BREAST_CANCER_10Q22_AMPLICON	9	5
NIKOLSKY_BREAST_CANCER_11Q12_Q14_AMPLICON	158	98
NIKOLSKY_BREAST_CANCER_12Q13_Q21_AMPLICON	46	34
NIKOLSKY_BREAST_CANCER_12Q24_AMPLICON	15	9
NIKOLSKY_BREAST_CANCER_14Q22_AMPLICON	14	12
NIKOLSKY_BREAST_CANCER_15Q26_AMPLICON	22	10
NIKOLSKY_BREAST_CANCER_16P13_AMPLICON	120	68
NIKOLSKY_BREAST_CANCER_16Q24_AMPLICON	53	32
NIKOLSKY_BREAST_CANCER_17P11_AMPLICON	10	9
NIKOLSKY_BREAST_CANCER_17Q11_Q21_AMPLICON	133	80
NIKOLSKY_BREAST_CANCER_17Q21_Q25_AMPLICON	335	200
NIKOLSKY_BREAST_CANCER_19P13_AMPLICON	5	5
NIKOLSKY_BREAST_CANCER_19Q13.1_AMPLICON	22	Rejected!
NIKOLSKY_BREAST_CANCER_19Q13.4_AMPLICON	7	Rejected!
NIKOLSKY_BREAST_CANCER_1Q21_AMPLICON	38	31
NIKOLSKY_BREAST_CANCER_1Q32_AMPLICON	13	10

NIKOLSKY_BREAST_CANCER_20P13_AMPLICON	8	7
NIKOLSKY_BREAST_CANCER_20Q11_AMPLICON	31	22
NIKOLSKY_BREAST_CANCER_20Q12_Q13_AMPLICON	149	85
NIKOLSKY_BREAST_CANCER_21Q22_AMPLICON	16	12
NIKOLSKY_BREAST_CANCER_22Q13_AMPLICON	17	8
NIKOLSKY_BREAST_CANCER_5P15_AMPLICON	26	20
NIKOLSKY_BREAST_CANCER_6P24_P22_AMPLICON	21	13
NIKOLSKY_BREAST_CANCER_7P15_AMPLICON	11	11
NIKOLSKY_BREAST_CANCER_7P22_AMPLICON	38	23
NIKOLSKY_BREAST_CANCER_7Q21_Q22_AMPLICON	76	44
NIKOLSKY_BREAST_CANCER_8P12_P11_AMPLICON	57	38
NIKOLSKY_BREAST_CANCER_8Q12_Q22_AMPLICON	132	86
NIKOLSKY_BREAST_CANCER_8Q23_Q24_AMPLICON	157	87
NIKOLSKY_MUTATED_AND_AMPLIFIED_IN_BREAST_CANCER	94	56
NIKOLSKY_OVERCONNECTED_IN_BREAST_CANCER	22	19
PACHER_TARGETS_OF_IGF1_AND_IGF2_UP	35	30
PECE_MAMMARY_STEM_CELL_DN	146	102
PECE_MAMMARY_STEM_CELL_UP	146	99
PEDERSEN_METASTASIS_BY_ERBB2_ISOFORM_1	46	37
PEDERSEN_METASTASIS_BY_ERBB2_ISOFORM_3	20	13
PEDERSEN_METASTASIS_BY_ERBB2_ISOFORM_4	110	77
PEDERSEN_METASTASIS_BY_ERBB2_ISOFORM_5	11	8
PEDERSEN_METASTASIS_BY_ERBB2_ISOFORM_6	29	20
PEDERSEN_METASTASIS_BY_ERBB2_ISOFORM_7	403	273
PEDERSEN_TARGETS_OF_611CTF_ISOFORM_OF_ERBB2	76	55
POOLA_INVASIVE_BREAST_CANCER_DN	134	97
POOLA_INVASIVE_BREAST_CANCER_UP	288	215
PUJANA_ATM_PCC_NETWORK	1442	Rejected!
PUJANA_BRCA1_PCC_NETWORK	1652	Rejected!
PUJANA_BRCA2_PCC_NETWORK	423	336
PUJANA_BRCA_CENTERED_NETWORK	117	103
PUJANA_BREAST_CANCER_LIT_INT_NETWORK	101	88
PUJANA_BREAST_CANCER_WITH_BRCA1_MUTATED_DN	9	6
PUJANA_BREAST_CANCER_WITH_BRCA1_MUTATED_UP	56	48
PUJANA_CHEK2_PCC_NETWORK	779	603
PUJANA_XPRSS_INT_NETWORK	168	138
RAY_TUMORIGENESIS_BY_ERBB2_CDC25A_DN	159	125
RAY_TUMORIGENESIS_BY_ERBB2_CDC25A_UP	104	80
RIGGINS_TAMOXIFEN_RESISTANCE_DN	220	174
RIGGINS_TAMOXIFEN_RESISTANCE_UP	66	58
RIZKI_TUMOR_INVASIVENESS_2D_DN	64	56
RIZKI_TUMOR_INVASIVENESS_2D_UP	69	56
RIZKI_TUMOR_INVASIVENESS_3D_DN	270	201
RIZKI_TUMOR_INVASIVENESS_3D_UP	210	163
ROYLANCE_BREAST_CANCER_16Q_COPY_NUMBER_DN	26	16
ROYLANCE_BREAST_CANCER_16Q_COPY_NUMBER_UP	63	33
SCHUETZ_BREAST_CANCER_DUCTAL_INVASIVE_DN	84	68
SCHUETZ_BREAST_CANCER_DUCTAL_INVASIVE_UP	351	266
SEITZ_NEOPLASTIC_TRANSFORMATION_BY_8P_DELETION_DN	30	27
SEITZ_NEOPLASTIC_TRANSFORMATION_BY_8P_DELETION_UP	73	54
SMID_BREAST_CANCER_BASAL_DN	701	476
SMID_BREAST_CANCER_BASAL_UP	648	493
SMID_BREAST_CANCER_ERBB2_DN	5	Rejected!
SMID_BREAST_CANCER_ERBB2_UP	147	98
SMID_BREAST_CANCER_LUMINAL_A_DN	18	15
SMID_BREAST_CANCER_LUMINAL_A_UP	84	63
SMID_BREAST_CANCER_LUMINAL_B_DN	564	412
SMID_BREAST_CANCER_LUMINAL_B_UP	172	121
SMID_BREAST_CANCER_NORMAL_LIKE_DN	6	5
SMID_BREAST_CANCER_NORMAL_LIKE_UP	476	348
SMID_BREAST_CANCER_RELAPSE_IN_BONE_DN	315	238
SMID_BREAST_CANCER_RELAPSE_IN_BONE_UP	97	64
SMID_BREAST_CANCER_RELAPSE_IN_BRAIN_DN	85	58
SMID_BREAST_CANCER_RELAPSE_IN_BRAIN_UP	39	37
SMID_BREAST_CANCER_RELAPSE_IN_LIVER_DN	10	10
SMID_BREAST_CANCER_RELAPSE_IN_LIVER_UP	6	6
SMID_BREAST_CANCER_RELAPSE_IN_LUNG_DN	37	25
SMID_BREAST_CANCER_RELAPSE_IN_LUNG_UP	21	18
SMID_BREAST_CANCER_RELAPSE_IN_PLEURA_DN	27	16
SMID_BREAST_CANCER_RELAPSE_IN_PLEURA_UP	6	6
SOTIRIOU_BREAST_CANCER_GRADE_1_VS_3_DN	52	35
SOTIRIOU_BREAST_CANCER_GRADE_1_VS_3_UP	151	118
SRC_UP.V1_DN	179	95
SRC_UP.V1_UP	188	107
STEIN_ESR1_TARGETS	85	70
STEIN_ESRRA_TARGETS	535	379
STEIN_ESRRA_TARGETS_DN	105	65
STEIN_ESRRA_TARGETS_RESPONSIVE_TO_ESTROGEN_DN	41	35
STEIN_ESRRA_TARGETS_RESPONSIVE_TO_ESTROGEN_UP	31	21
STEIN_ESRRA_TARGETS_UP	388	286
STEIN_ESTROGEN_RESPONSE_NOT_VIA_ESRRA	18	16

STREICHER_LSM1_TARGETS_DN	19	15
STREICHER_LSM1_TARGETS_UP	44	37
SUBTIL_PROGESTIN_TARGETS	36	29
TAVAZOIE_METASTASIS	108	65
THEODOROU_MAMMARY_TUMORIGENESIS	31	24
THILLAINADESAN_ZNF217_TARGETS_DN	9	6
THILLAINADESAN_ZNF217_TARGETS_UP	44	31
TURASHVILI_BREAST_CARCINOMA_DUCTAL_VS_LOBULAR_DN	7	Rejected!
TURASHVILI_BREAST_CARCINOMA_DUCTAL_VS_LOBULAR_UP	21	13
TURASHVILI_BREAST_DUCTAL_CARCINOMA_VS_DUCTAL_NORMAL_DN	198	132
TURASHVILI_BREAST_DUCTAL_CARCINOMA_VS_DUCTAL_NORMAL_UP	44	35
TURASHVILI_BREAST_DUCTAL_CARCINOMA_VS_LOBULAR_NORMAL_DN	69	45
TURASHVILI_BREAST_DUCTAL_CARCINOMA_VS_LOBULAR_NORMAL_UP	73	57
TURASHVILI_BREAST_LOBULAR_CARCINOMA_VS_DUCTAL_NORMAL_DN	91	63
TURASHVILI_BREAST_LOBULAR_CARCINOMA_VS_DUCTAL_NORMAL_UP	69	53
TURASHVILI_BREAST_LOBULAR_CARCINOMA_VS_LOBULAR_NORMAL_DN	74	57
TURASHVILI_BREAST_LOBULAR_CARCINOMA_VS_LOBULAR_NORMAL_UP	94	66
TURASHVILI_BREAST_NORMAL_DUCTAL_VS_LOBULAR_DN	7	Rejected!
TURASHVILI_BREAST_NORMAL_DUCTAL_VS_LOBULAR_UP	68	34
V$AP2_Q3	251	194
V$AP2_Q6	258	184
V$AP2_Q6_01	272	192
V$AP2GAMMA_01	250	179
V$HMGIY_Q6	248	174
V$STAT1_02	252	174
V$STAT1_03	248	174
VANTVEER_BREAST_CANCER_BRCA1_DN	44	29
VANTVEER_BREAST_CANCER_BRCA1_UP	36	30
VANTVEER_BREAST_CANCER_ESR1_DN	240	185
VANTVEER_BREAST_CANCER_ESR1_UP	167	111
VANTVEER_BREAST_CANCER_METASTASIS_DN	121	84
VANTVEER_BREAST_CANCER_METASTASIS_UP	56	34
VANTVEER_BREAST_CANCER_POOR_PROGNOSIS	55	32
VETTER_TARGETS_OF_PRKCA_AND_ETS1_DN	16	10
VETTER_TARGETS_OF_PRKCA_AND_ETS1_UP	16	12
WANG_METASTASIS_OF_BREAST_CANCER	15	9
WANG_METASTASIS_OF_BREAST_CANCER_ESR1_DN	30	22
WANG_METASTASIS_OF_BREAST_CANCER_ESR1_UP	22	17
WANG_METHYLATED_IN_BREAST_CANCER	35	32
WILLIAMS_ESR1_TARGETS_DN	6	Rejected!
WILLIAMS_ESR1_TARGETS_UP	26	22
WILLIAMS_ESR2_TARGETS_DN	11	8
WILLIAMS_ESR2_TARGETS_UP	28	23
WILSON_PROTEASES_AT_TUMOR_BONE_INTERFACE_DN	5	5
WILSON_PROTEASES_AT_TUMOR_BONE_INTERFACE_UP	21	19
WINTER_HYPOXIA_DN	52	38
WINTER_HYPOXIA_METAGENE	242	198
WINTER_HYPOXIA_UP	92	61
WONG_MITOCHONDRIA_GENE_MODULE	217	178
WONG_PROTEASOME_GENE_MODULE	49	42
ZHANG_BREAST_CANCER_PROGENITORS_DN	145	104
ZHANG_BREAST_CANCER_PROGENITORS_UP	425	308
ZHU_SKIL_TARGETS_DN	9	9
ZHU_SKIL_TARGETS_UP	20	17
ZUCCHI_METASTASIS_DN	44	36
ZUCCHI_METASTASIS_UP	43	36
