# Supplementary material for: Analysis of high fat diet induced genes during mammary gland development: identifying role players in poor prognosis of breast cancer
Source: BMC Res Notes. 2014 Aug 18;7:543. doi: 10.1186/1756-0500-7-543 (PMC4153917; doi:10.1186/1756-0500-7-543)
Supplement: Supplementary file 2 — Additional file 2: 53 luminal breast cancer gene sets that were utilized in GSEA. This excel file details the 53 breast cancer gene sets from the Molecular Signatures Database that were utilized for gene set enrichment analysis with the HFD microarray data. NAME denotes the set name, original size denotes the number of genes in the curated gene set, after restricting to data set denotes the number of genes that were found on both the gene list and the HFD microarray data set, and status denotes whether the gene list was included in the analysis or not. A small number of gene lists were not included in the analysis, if there were not enough genes. (PDF 184 KB) [file 13104_2014_3097_MOESM2_ESM.pdf]

**Additional File 2. 53 Luminal Breast Cancer Gene Sets that were Utilized in GSEA**

| NAME | ORIGINAL SIZE | AFTER RESTRICTING TO DATASET | STATUS |
|---|---|---|---|
| BERTUCCI_MEDULLARY_VS_DUCTAL_BREAST_CANCER_DN | 169 | 119 | |
| BERTUCCI_MEDULLARY_VS_DUCTAL_BREAST_CANCER_UP | 206 | 142 | |
| CHARAFE_BREAST_CANCER_BASAL_VS_MESENCHYMAL_DN | 50 | 35 | |
| CHARAFE_BREAST_CANCER_BASAL_VS_MESENCHYMAL_UP | 121 | 76 | |
| CHARAFE_BREAST_CANCER_LUMINAL_VS_BASAL_DN | 455 | 337 | |
| CHARAFE_BREAST_CANCER_LUMINAL_VS_BASAL_UP | 380 | 230 | |
| CHARAFE_BREAST_CANCER_LUMINAL_VS_MESENCHYMAL_DN | 460 | 325 | |
| CHARAFE_BREAST_CANCER_LUMINAL_VS_MESENCHYMAL_UP | 450 | 293 | |
| FARMER_BREAST_CANCER_APOCRINE_VS_BASAL | 330 | 245 | |
| FARMER_BREAST_CANCER_APOCRINE_VS_LUMINAL | 326 | 235 | |
| FARMER_BREAST_CANCER_BASAL_VS_LULMINAL | 330 | 241 | |
| FARMER_BREAST_CANCER_CLUSTER_1 | 44 | 25 | |
| FARMER_BREAST_CANCER_CLUSTER_2 | 33 | 30 | |
| FARMER_BREAST_CANCER_CLUSTER_4 | 16 | 14 | |
| FARMER_BREAST_CANCER_CLUSTER_5 | 19 | 18 | |
| FARMER_BREAST_CANCER_CLUSTER_6 | 16 | 14 | |
| FARMER_BREAST_CANCER_CLUSTER_7 | 20 | 13 | |
| FARMER_BREAST_CANCER_CLUSTER_8 | 7 | | Rejected! |
| FINETTI_BREAST_CANCER_KINOME_GREEN | 16 | 15 | |
| FINETTI_BREAST_CANCER_KINOME_RED | 16 | 15 | |
| FINETTI_BREAST_CANCERS_KINOME_BLUE | 21 | 17 | |
| FINETTI_BREAST_CANCERS_KINOME_GRAY | 15 | 13 | |
| HOWLIN_CITED1_TARGETS_1_DN | 37 | 29 | |
| HOWLIN_CITED1_TARGETS_1_UP | 35 | 25 | |
| HOWLIN_CITED1_TARGETS_2_DN | 17 | 16 | |
| HOWLIN_CITED1_TARGETS_2_UP | 17 | 16 | |
| HOWLIN_PUBERTAL_MAMMARY_GLAND | 69 | 60 | |
| HUPER_BREAST_BASAL_VS_LUMINAL_DN | 59 | 43 | |
| HUPER_BREAST_BASAL_VS_LUMINAL_UP | 54 | 38 | |
| LIM_MAMMARY_LUMINAL_MATURE_DN | 99 | 82 | |
| LIM_MAMMARY_LUMINAL_MATURE_UP | 116 | 85 | |
| LIM_MAMMARY_LUMINAL_PROGENITOR_DN | 14 | 11 | |
| LIM_MAMMARY_LUMINAL_PROGENITOR_UP | 58 | 49 | |
| LIM_MAMMARY_STEM_CELL_DN | 428 | 308 | |
| LIM_MAMMARY_STEM_CELL_UP | 489 | 372 | |
| SMID_BREAST_CANCER_BASAL_DN | 701 | 476 | |
| SMID_BREAST_CANCER_BASAL_UP | 648 | 493 | |
| SMID_BREAST_CANCER_ERBB2_DN | 5 | | Rejected! |
| SMID_BREAST_CANCER_ERBB2_UP | 147 | 98 | |
| SMID_BREAST_CANCER_LUMINAL_A_DN | 18 | 15 | |
| SMID_BREAST_CANCER_LUMINAL_A_UP | 84 | 63 | |
| SMID_BREAST_CANCER_LUMINAL_B_DN | 564 | 412 | |
| SMID_BREAST_CANCER_LUMINAL_B_UP | 172 | 121 | |
| SMID_BREAST_CANCER_NORMAL_LIKE_DN | 6 | 5 | |
| SMID_BREAST_CANCER_NORMAL_LIKE_UP | 476 | 348 | |
| SMID_BREAST_CANCER_RELAPSE_IN_BONE_DN | 315 | 238 | |
| SMID_BREAST_CANCER_RELAPSE_IN_BONE_UP | 97 | 64 | |
| SMID_BREAST_CANCER_RELAPSE_IN_BRAIN_DN | 85 | 58 | |
| SMID_BREAST_CANCER_RELAPSE_IN_BRAIN_UP | 39 | 37 | |
| SMID_BREAST_CANCER_RELAPSE_IN_LIVER_DN | 10 | 10 | |
| SMID_BREAST_CANCER_RELAPSE_IN_LIVER_UP | 6 | 6 | |
| SMID_BREAST_CANCER_RELAPSE_IN_LUNG_DN | 37 | 25 | |
| SMID_BREAST_CANCER_RELAPSE_IN_LUNG_UP | 21 | 18 | |
| SMID_BREAST_CANCER_RELAPSE_IN_PLEURA_DN | 27 | 16 | |
| SMID_BREAST_CANCER_RELAPSE_IN_PLEURA_UP | 6 | 6 | |
