# Supplementary figures and images for: Analysis of high fat diet induced genes during mammary gland development: identifying role players in poor prognosis of breast cancer
Source: BMC Res Notes. 2014 Aug 18;7:543. doi: 10.1186/1756-0500-7-543 (PMC4153917; doi:10.1186/1756-0500-7-543)

Subtype

|            |      |           |           |             |
|------------|------|-----------|-----------|-------------|
| Basal-like | HER2 | Luminal A | Luminal B | Normal-like |
|------------|------|-----------|-----------|-------------|

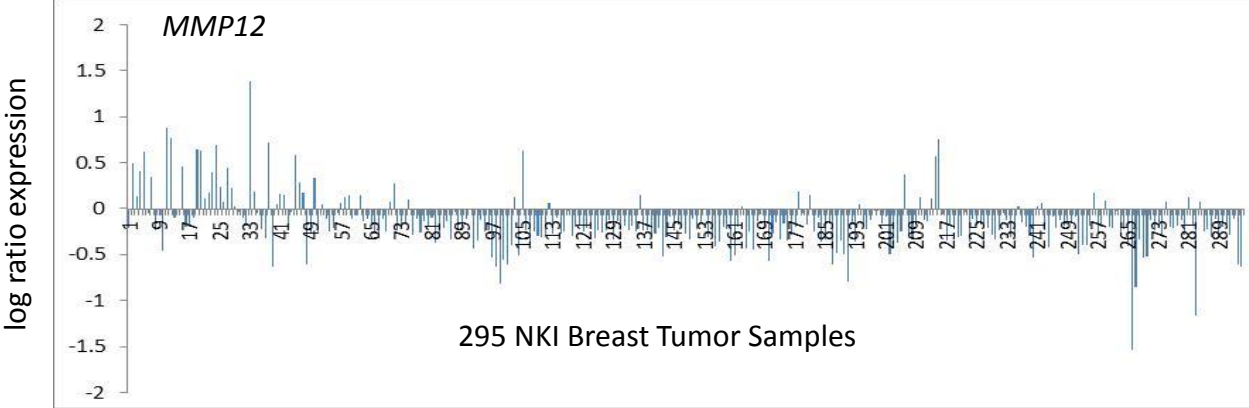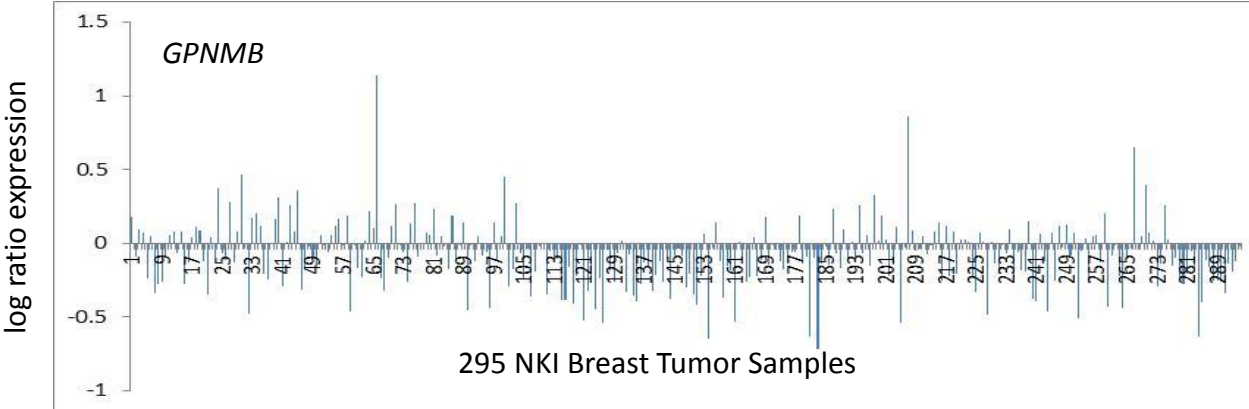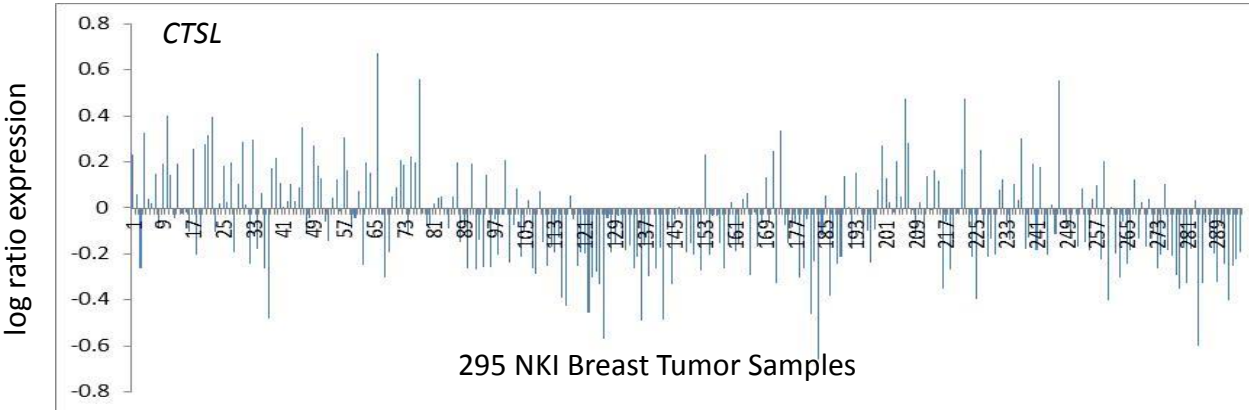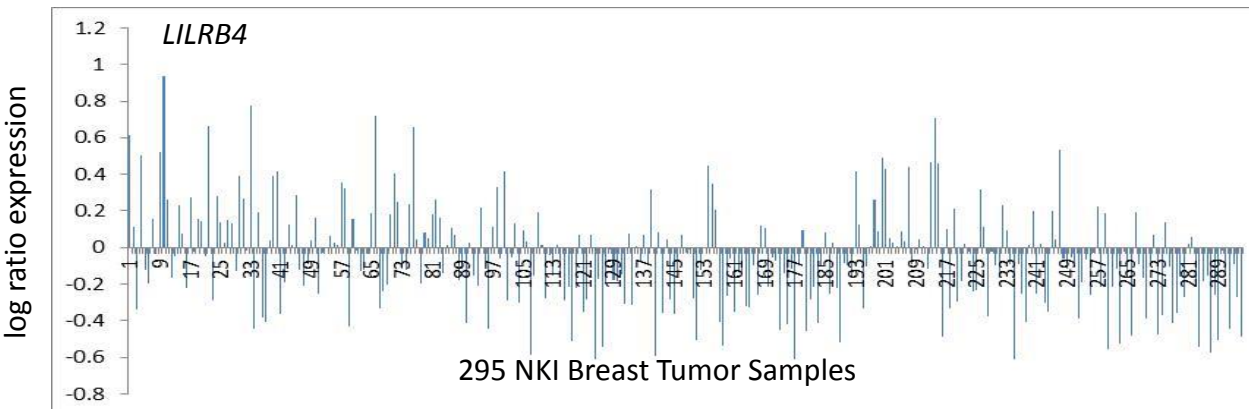

Supplement: Supplementary file 3 — Additional file 3: Poor prognosis associated HFD-induced genes MMP12, GPNMB, CTSL and LILRB4 are highly expressed in basal-like BC. Depicted are histograms with log ration expression values for the indicated gene for each tumor (from the 295 NKI breast tumor dataset). Tumors are grouped together based on subtype. These subtypes are indicated at the top of the figure: Basal-like tumors are 1–46, HER2 are 47–95, Luminal A are 96–183, Luminal B are 184–264 and Normal-like tumors are 265–295. (PDF 326 KB) [file 13104_2014_3097_MOESM3_ESM.pdf]

Subtype

|            |      |           |           |             |
|------------|------|-----------|-----------|-------------|
| Basal-like | HER2 | Luminal A | Luminal B | Normal-like |
|------------|------|-----------|-----------|-------------|

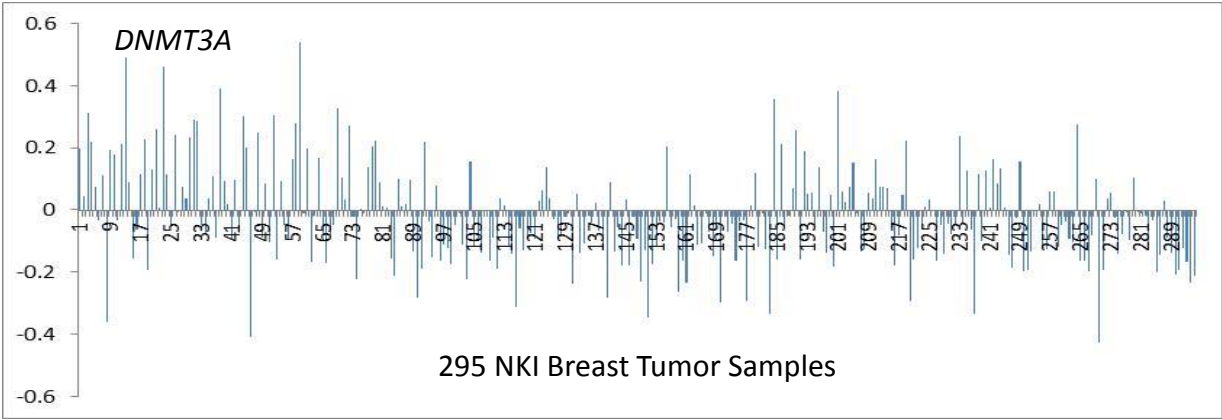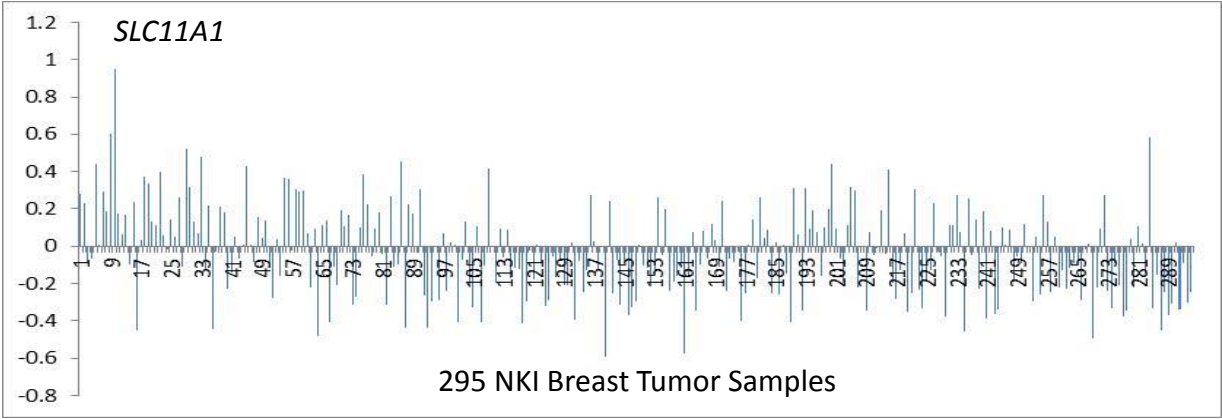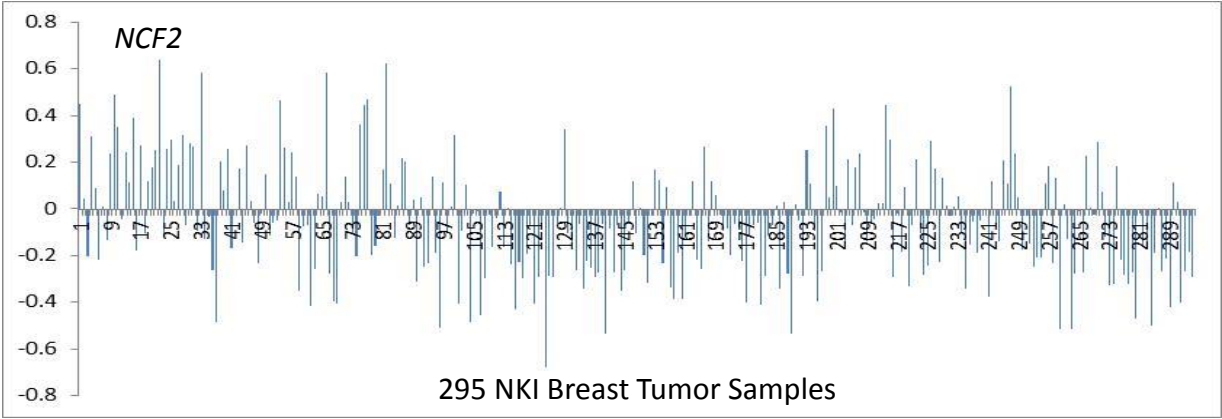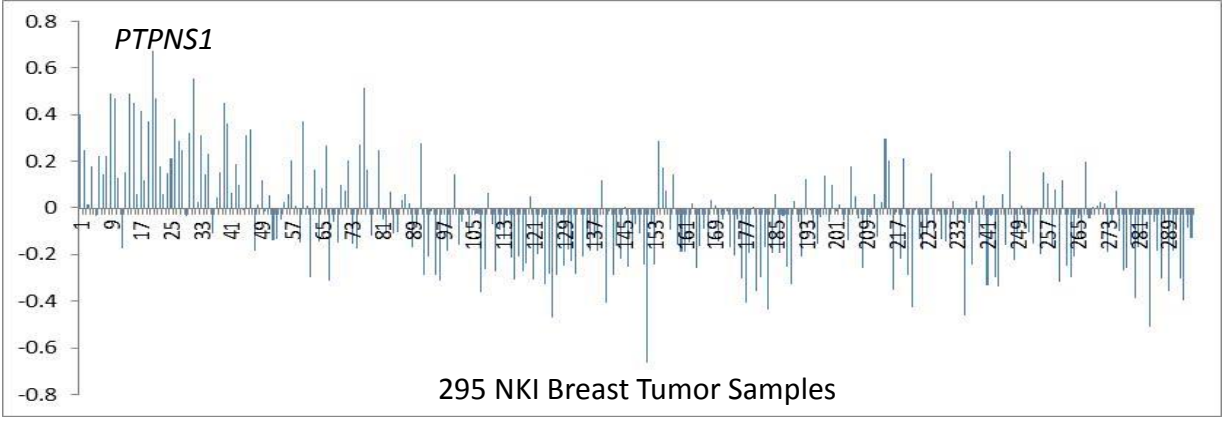

Supplement: Supplementary file 4 — Additional file 4: Poor prognosis associated HFD-induced genes DNMT3A, SLC11A1, NCF2 and PTPNS1 are highly expressed in basal-like BC. Depicted are histograms with log ration expression values for the indicated gene for each tumor (from the 295 NKI breast tumor dataset). Tumors are grouped together based on subtype. These subtypes are indicated at the top of the figure: Basal-like tumors are 1–46, HER2 are 47–95, Luminal A are 96–183, Luminal B are 184–264 and Normal-like tumors are 265–295. (PDF 335 KB) [file 13104_2014_3097_MOESM4_ESM.pdf]
